# Pulmonary tumor thrombotic microangiopathy in occult early gastric cancer that was undetectable on upper endoscopy: a case report and review of similar cases

**DOI:** 10.1186/s12876-021-02009-8

**Published:** 2021-11-10

**Authors:** Naoki Kawakami, Tomohiro Moriya, Rina Kato, Kentaro Nakamura, Hiroaki Saito, Yoko Wakai, Kazuhito Saito, Mai Sakashita

**Affiliations:** 1 Department of Respiratory Medicine, Tsuchiura Kyodo General Hospital, 4-1-1 Otsuno, Tsuchiura, Ibaraki 300-0028 Japan; 2 Department of Pathology, Tsuchiura Kyodo General Hospital, 4-1-1 Otsuno, Tsuchiura, Ibaraki 300-0028 Japan

**Keywords:** Pulmonary tumor thrombotic microangiopathy, Early gastric cancer, Signet ring cell carcinoma, Endoscopy, Imatinib

## Abstract

**Background:**

Pulmonary tumor thrombotic microangiopathy (PTTM), a rare manifestation of metastatic cancer with poor prognosis, is characterized by subacute/acute fatal pulmonary hypertension. The main cause of PTTM is gastric cancer, and cases of early gastric cancer confirmed using autopsy have been reported. Moreover, several cases of early gastric cancer that are undetectable on endoscopy or macroscopic postmortem examination have been reported.

**Case presentation:**

A previously healthy 50-year-old man presented with progressive dyspnea and cough for 1 month. Echocardiography suggested pulmonary hypertension. Computed tomography revealed diffuse lymphadenopathy, whereas blood work revealed an elevation in several serum tumor marker levels. Despite normal upper endoscopic findings, a presumptive diagnosis of PTTM due to gastric cancer was made based on pathological findings of cervical lymph node biopsy, which indicated signet ring cell carcinoma. Imatinib and tegafur/gimeracil/oteracil plus oxaliplatin therapy were started on day 7. The patient’s condition was initially stable. However, his symptoms suddenly progressed, and the patient died on day 8. Macroscopic postmortem examination revealed no abnormal gastric wall findings. Microscopically, PTTM was confirmed, and multiple serial sections of the stomach revealed early gastric cancer.

**Conclusions:**

Despite normal endoscopic findings, micro-occult gastric cancer can lead to PTTM. Physicians should be aware of this disease presentation. Taking prompt action is needed when PTTM is suspected, even if the patient appears stable.

**Supplementary Information:**

The online version contains supplementary material available at 10.1186/s12876-021-02009-8.

## Background

Pulmonary tumor thrombotic microangiopathy (PTTM) is a rare manifestation of metastatic cancer and has a poor prognosis. PTTM is pathologically characterized by fibrocellular intimal proliferation and focal hypercoagulability that cause pulmonary artery obstruction, leading to fatal pulmonary hypertension. The clinical course is characterized by rapidly progressive dyspnea, often resulting in sudden death. Among cancer patients, the incidence of PTTM, as confirmed by autopsy, has been reported to range from 1 to 3%, with gastric cancer being the dominant cause [1, 2]. Cases of PTTM in early gastric cancer confirmed on autopsy have been previously reported [3–6]. Moreover, occasional cases of early or micro-occult gastric cancer are undetectable on endoscopy or macroscopic postmortem examination [7–9]. Herein, we report a case of PTTM with an antemortem presumptive diagnosis of micro-occult gastric cancer that was undetectable using endoscopy but was confirmed using postmortem microscopic examination. A review of similar reported cases is also included.

## Case presentation

A previously healthy 50-year-old man who was a smoker was referred to the emergency department with progressive dry cough and dyspnea for 1 month. He had been prescribed antiallergic and antitussive medications by a family doctor; however, they were ineffective. The patient had no family history of cardiopulmonary disease or malignancy. Physical examination and blood tests in the emergency department were non-diagnostic. Electrocardiography revealed negative T waves, and echocardiography was suggestive of pulmonary hypertension. Enhanced computed tomography (CT) revealed no apparent pulmonary embolism. He was prescribed a bronchodilator and corticosteroid under a diagnosis of cor pulmonale due to chronic obstructive pulmonary disease; however, his symptoms did not alleviate. The patient was admitted to our hospital two days later.

His vital signs on admission were as follows: oxygen saturation, 94%; pulse rate, 100 beats per minute; body temperature, 37.1 °C; blood pressure, 130/93 mmHg; and respiratory rate, 18 breaths per minute. Physical examination revealed right cervical lymphadenopathy, with the affected lymph node measuring 10 mm in size. Laboratory findings were as follows: 95.7 (normal: < 18.4) pg/mL of brain natriuretic peptide, 6.9 (normal: < 1.0) µg/mL of D-dimer, 134 (normal: 124–222) U/L of lactate dehydrogenase, 1.77 (normal: < 0.15) mg/dL of C-reactive protein, 8.7 (normal: < 5.0) ng/mL of carcinoembryonic antigen, and 19.4 (normal: < 2.1) ng/mL of cytokeratin-19 fragment.

**Fig. 1:**
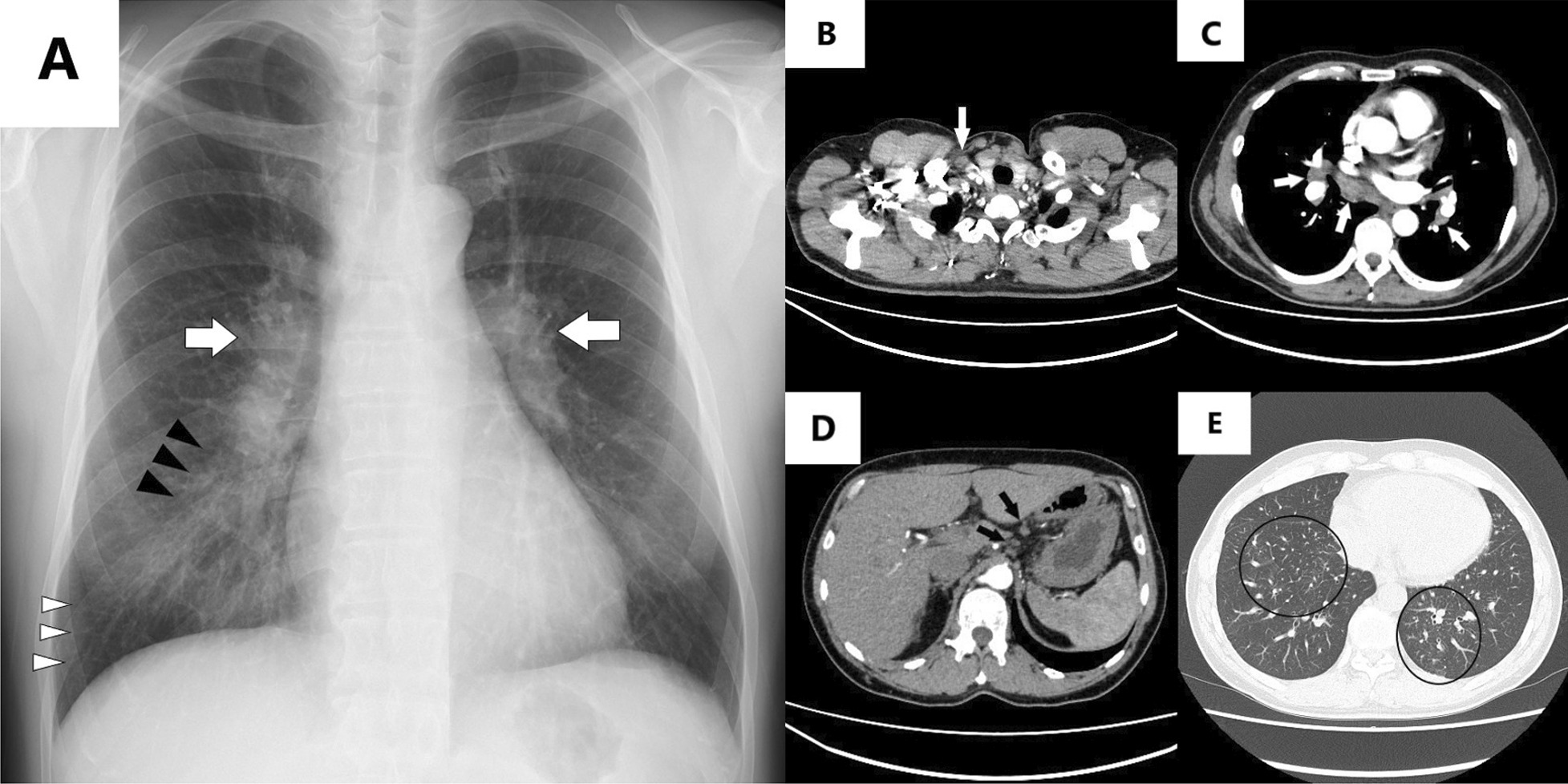
Radiographic findings on admission. **A** Chest radiography shows bilateral hilar lymphadenopathy (white arrows), ground-glass opacities (black arrows), and Kerley B lines (white arrowheads) in the right lung. Enhanced computed tomography shows **B** right cervical lymphadenopathy (white arrow), **C** hilar/mediastinal lymphadenopathy (white arrows), and **D** upper abdominal lymphadenopathy (black arrows). **E** Septal thickening and ground-glass opacities are observed in the lower lung lobes (black circles)

Chest radiography revealed bilateral hilar lymphadenopathy (Fig. 1A). Enhanced CT showed no pulmonary embolism or deep vein thrombosis; however, right cervical, bilateral hilar/mediastinal, and upper abdominal lymphadenopathy was observed (Fig. 1B–D). Moreover, septal thickening and ground-glass opacities were predominantly detected in the lower lung lobes (Fig. 1E).

**Fig. 2:**
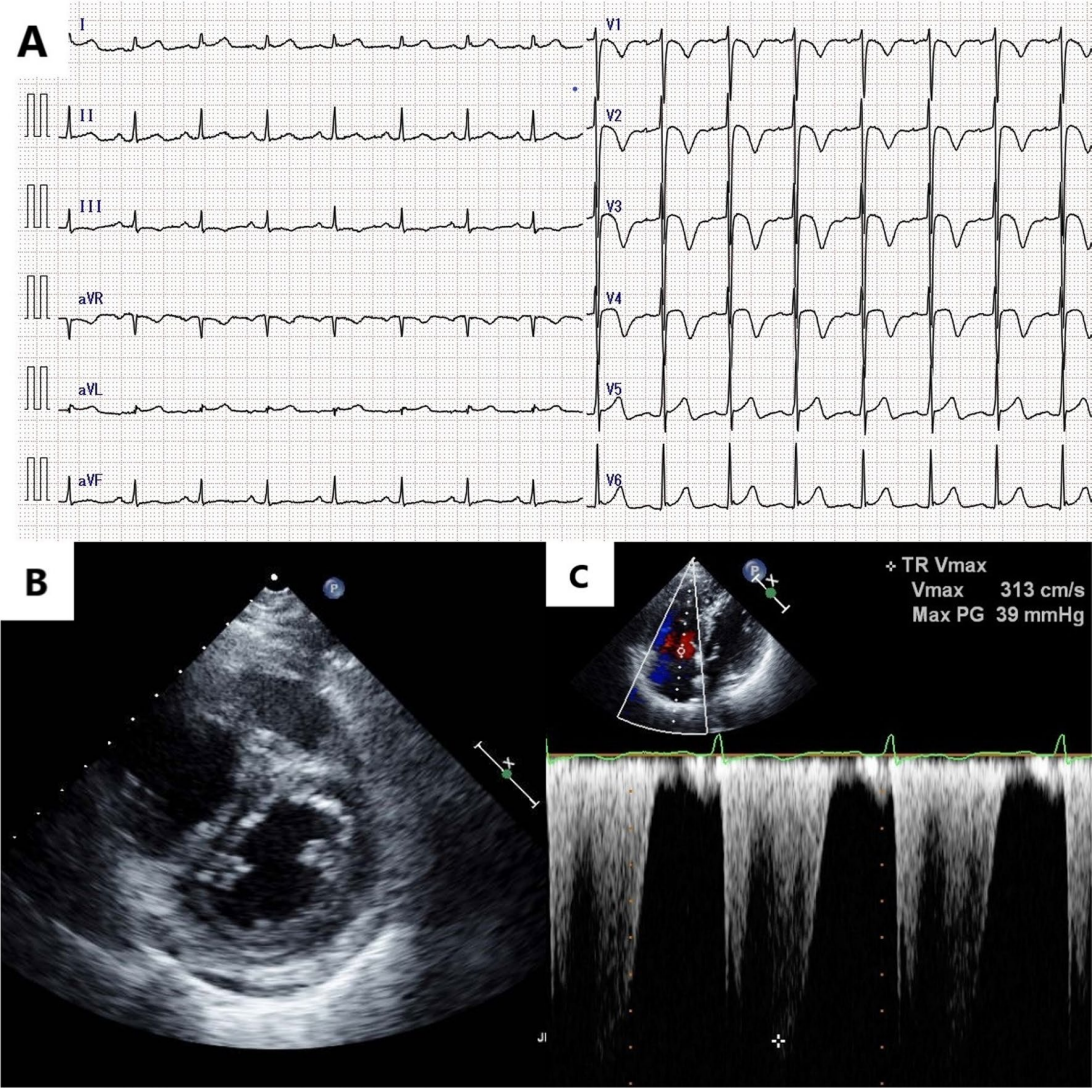
Physiological examination findings on admission. **A** Electrocardiography shows negative T waves in precordial leads. **B** Echocardiography shows D-shape of the left ventricle in systolic phase and **C** elevated tricuspid regurgitation pressure gradient at 39 mmHg

Electrocardiography showed negative T waves in precordial leads (Fig. 2A), and echocardiography indicated pulmonary hypertension with normal left ventricular function (Fig. 2B, C).

**Fig. 3:**
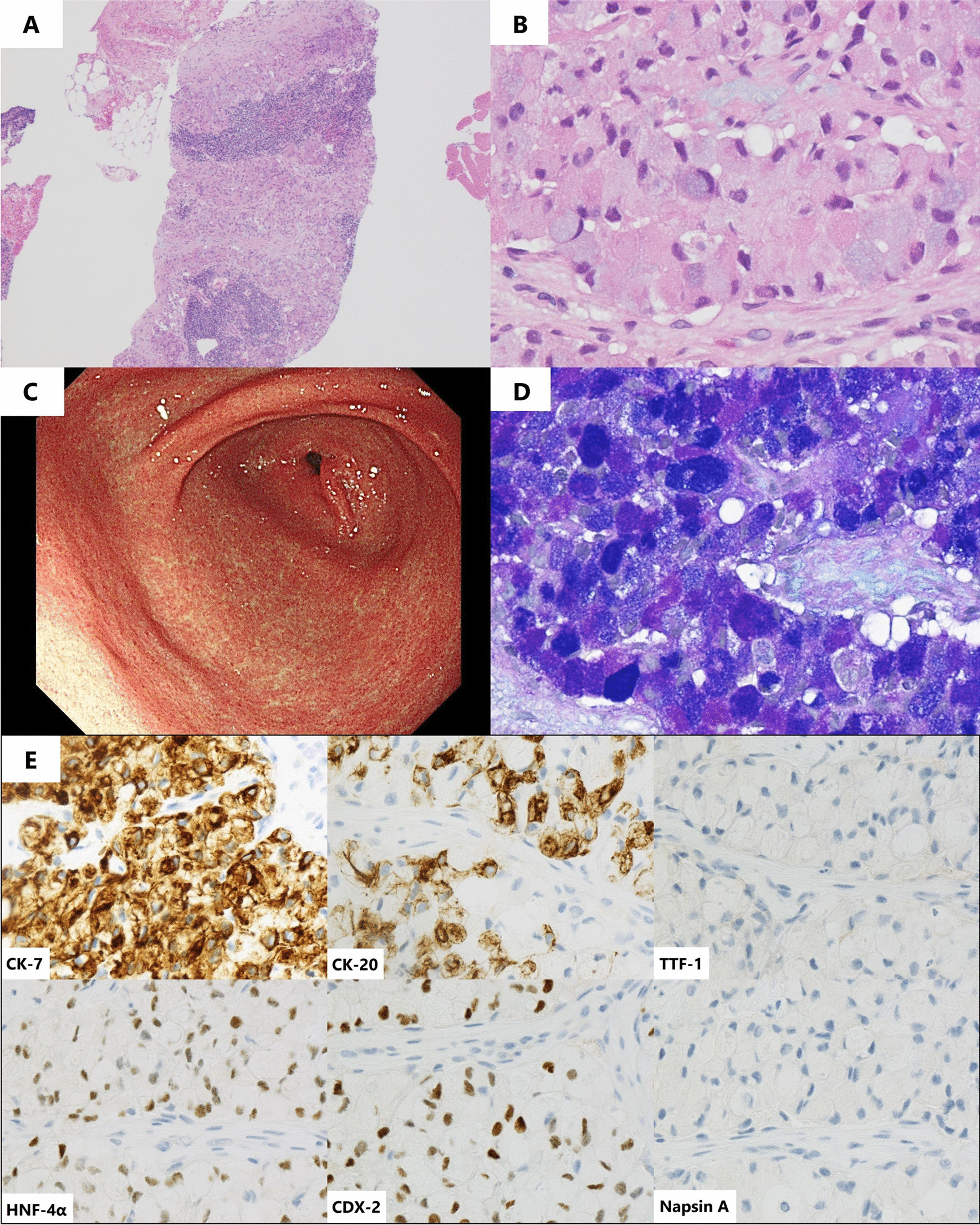
Pathological findings of right cervical lymph node biopsy. **A** Pathological findings of right cervical lymph node biopsy with hematoxylin and eosin staining indicate tumor cells in the specimen (×40) as well as **B** tumor cells with a signet ring cell feature (×400). **C** Upper endoscopy shows no abnormal findings in the pyloric region of the stomach. **D** Lymph node biopsy with Alcian blue-periodic acid-Schiff staining shows mucin-abundant tumor cells (×400). **E** Immunohistochemical staining shows positive cytokeratin (CK)-7, positive CK-20, negative thyroid transcription factor-1 (TTF-1), positive hepatocyte nuclear factor-4α (HNF-4α), positive caudal-type homeobox-2 (CDX-2), and negative napsin A (×400)

We suspected PTTM because of the subacute course, pulmonary hypertension without apparent etiology, elevated tumor marker levels, and diffuse lymphadenopathy. The primary cancer origin could not be determined using CT or tumor markers. Echo-guided needle biopsy of the right cervical lymph node was performed on day 1. Stamp cytology of the biopsy revealed atypical cells of epithelial origin. On day 2, a transbronchial lung biopsy was performed. On day 3, an interim report regarding the pathological findings of cervical lymph node biopsy revealed adenocarcinoma with signet ring cell carcinoma (SRCC) (Fig. 3A, B). Therefore, gastric cancer was suspected; however, upper endoscopy performed by a trained gastrointestinal endoscopist showed no mucosal abnormalities and a fully distensible gastric wall (Fig. 3C). Colonoscopy results were also normal. On day 4, the patient started receiving edoxaban (60 mg/day) for probable hypercoagulability due to PTTM. On day 5, the reported results of cervical lymph node biopsy with immunohistochemical staining were consistent with those of gastric cancer (Fig. 3D, E). The pathological findings of transbronchial lung biopsy were unremarkable. At this time, the patient’s status was stable without any apparent changes.

On day 6, his dyspnea mildly progressed, and oxygen delivery was initiated at 2 L/min via a nasal cannula. On day 7, after consultation with gastroenterologists at our institution, administration of imatinib (200 mg/day), prednisolone (30 mg/day), and tegafur/gimeracil/oteracil plus oxaliplatin (SOX) therapy was initiated. On day 8, the patient’s hypoxia suddenly worsened, and he subsequently died.

**Fig. 4:**
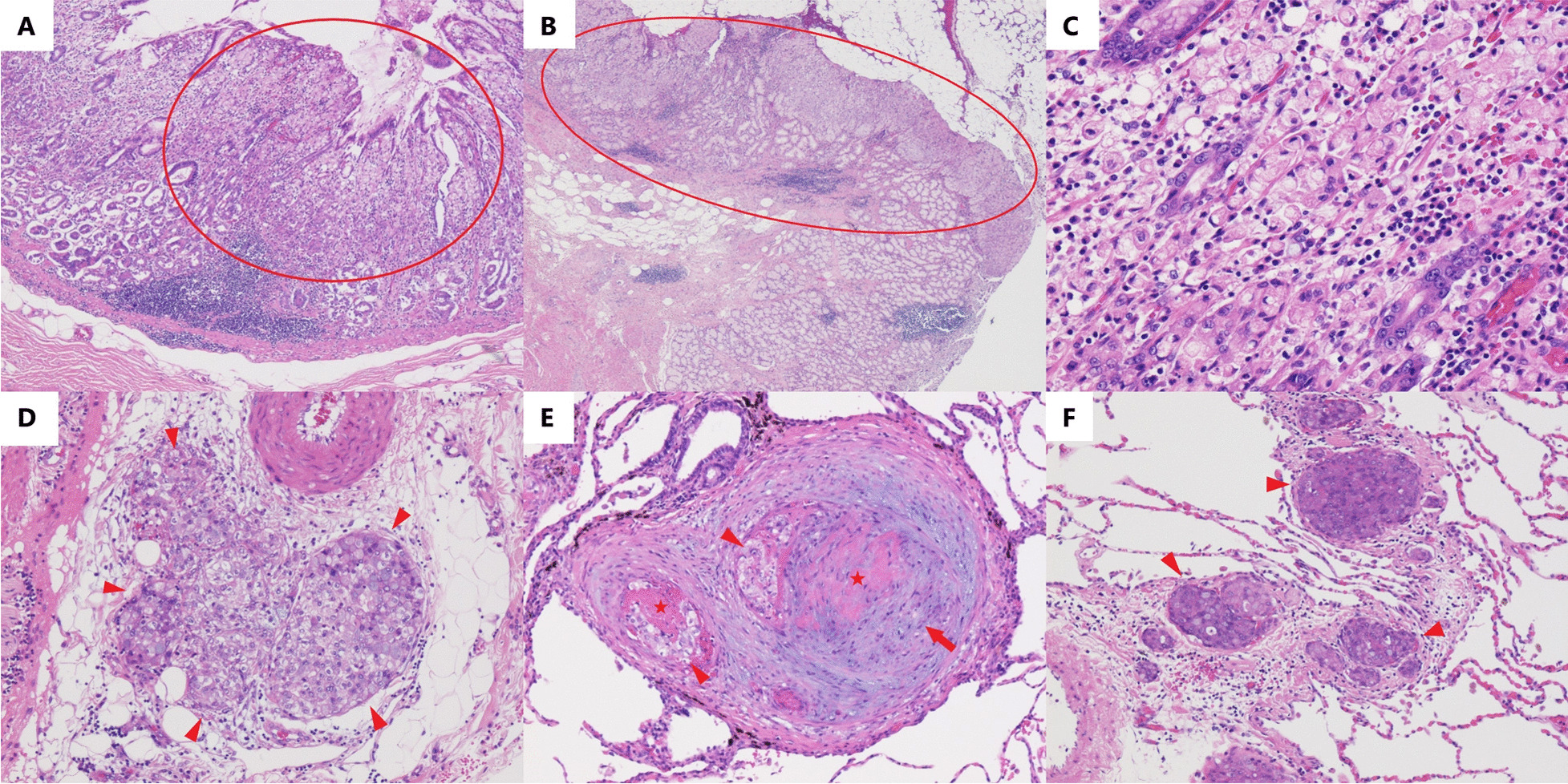
Postmortem pathological findings. **A**, **B** Postmortem pathological findings of hematoxylin and eosin staining of the stomach show two gastric cancer lesions in the pyloric region (×40 and ×20, respectively). These two lesions are within the lamina propria (circles). **C** The magnified cancer lesion shows signet ring cell carcinoma (×200). **D** Lymphovascular invasion is observed in the subserosa (arrowheads) (×100). **E** Pathological findings of the lung show tumor emboli (arrowheads), thickened epithelial cells (arrow), fibrin thrombi (stars) of small pulmonary arteries (×100), and **F** carcinomatous lymphangiomatosis (arrowheads) (×100)

An autopsy was performed, and macroscopic examination showed no abnormalities in the gastric wall (Additional file 1a); however, lymphadenopathy surrounding the stomach was detected. Macroscopic pulmonary thromboembolism was not observed. Multiple serial sections of the whole stomach were prepared. Microscopically, two gastric cancer lesions of pure SRCC that measured 15 $\times \hspace{0.17em}$12 mm and 8 $\times \hspace{0.17em}$8 mm were identified in the pyloric region (Fig. 4A–C and Additional file 1b and c). Although these lesions were within the lamina propria, multiple lymphovascular invasions separate from the two superficial lesions were observed in the layers from the submucosa to the subserosa (Fig. 4D). Multiple lymph node metastases surrounded the stomach. No findings of *Helicobacter pylori*-associated gastritis were observed. Moreover, tumor emboli and thickened epithelial cells of the small pulmonary arteries were consistent with PTTM (Fig. 4E). Carcinomatous lymphangiomatosis was also observed (Fig. 4F). Examination of organs other than those mentioned above showed no tumor cell in the viscera or vascular system.

The final diagnosis was PTTM in occult early gastric cancer that was undetectable on upper endoscopy.

## Discussion and Conclusions

PTTM is a rare manifestation of metastatic cancer with a poor prognosis. It is pathologically characterized by fibrocellular intimal proliferation and focal thrombosis that cause pulmonary artery obstruction, frequently leading to fatal pulmonary hypertension. The clinical course is characterized by rapidly progressive dyspnea and, often, sudden death. A typical presentation of PTTM is acute/subacute pulmonary hypertension without an apparent etiology. Among cancer patients, the incidence of PTTM, as confirmed by autopsy, has been reported to range from 1.4 to 3.3%, and most were gastric cancer cases [1, 2].

Differential diagnoses of PTTM include chronic thromboembolic pulmonary hypertension (CTEPH) or pulmonary arterial hypertension, which can be due to pulmonary veno-occlusive disease following chemotherapy. All these conditions have a similar presentation as pulmonary hypertension without an apparent etiology; nonetheless, CTEPH and pulmonary arterial hypertension are chronic, while PTTM usually presents as an acute/subacute disease [1, 2].

Our patient complained of cough for 1 month before presentation. Cough in PTTM is generally dry and is observed in 85% of cases. In some cases, it can precede dyspnea by months [2].

In our case, elevation in tumor marker levels and diffuse lymphadenopathy were diagnostic clues. We made a presumptive diagnosis of PTTM in gastric cancer based on pathological findings of cervical lymph node biopsy, lymphadenopathy in the thorax and upper abdomen, and pulmonary hypertension of otherwise unknown etiology. Mediastinal/hilar lymphadenopathy is common in PTTM of gastric cancer [2], and in our case, abdominal lymphadenopathy near the stomach suggested gastric cancer. Antemortem definitive pathological diagnosis of PTTM is made using transbronchial or surgical lung biopsy. However, lung biopsy is usually impossible because of the unstable cardiopulmonary status. Blood sampling using a right heart catheter can be helpful for diagnosing primary cancer and tumor emboli. However, like lung biopsy, a right heart catheter is often inapplicable because of rapid clinical deterioration. Clinically, PTTM is presumptively diagnosed based on the combination of acute/subacute pulmonary hypertension with the presence of cancer. However, because of its rarity, PTTM is not initially suspected in patients without a history of cancer. Thus, antemortem diagnosis of PTTM is challenging both pathologically and clinically [1, 2].

We detected occult early gastric cancer with microscopic observation using multiple serial step sections of the whole stomach but not using endoscopy. Cases of PTTM in early gastric cancer confirmed using autopsy have been previously reported [3–6]. Moreover, some early or micro-occult gastric cancer cases cannot be detected by endoscopy or macroscopic postmortem examination [7–9]. We searched the PubMed database for the terms “Pulmonary Tumor Thrombotic Microangiopathy,” “Pulmonary Tumour Thrombotic Microangiopathy,” and “PTTM” to identify cases of PTTM in early gastric cancer or micro-occult gastric cancer that were undetectable on endoscopy or macroscopic postmortem examination. Twelve cases of PTTM in early or micro-occult gastric cancer that contained descriptions of pathological invasion depth were identified (Table 1) [3–9]. The age distribution of the patients was 17–75 years (median age, 51 years). Three patients were under 50 years, but a familial history of cancer was not indicated in all cases, including our case. Eight cases, including our patient, were men and five cases were women. The maximum diameter of the cancer lesion was 30 mm. Macroscopic latency was suspected to be due to small mucosal cancer lesions (1.8–15 mm) or flat lesions.

**Table 1:** PTTM cases of early or micro-occult gastric cancer undetectable on endoscopy or macroscopic postmortem examination

Case no	References	Age and sex	Type	Maximum diameter (mm)	Invasion depth	Endoscopic findings	Initial pathological method for confirming cancer	Initial method for identifying cancer in the stomach	Other clinical findings	Pharmacotherapy
1	Sato [3]	50M	Sig + tub	30	Early (SM)	An ulcerated lesion	Biopsy of the stomach	Endoscopy	Metastasis of cervical lymph nodes, skin, and multiple bones	5-FU + MMC + CDDP (death on the 86th hospital day)
2	Igarashi [4, 5]	29F	Sig	20	Early (M)	Not performed	Autopsy	Autopsy (macroscopy)	18 weeks of pregnancy; metastasis of multiple lymph node, bone marrow, and ovaries	AC (death on the 5th hospital day)
3	Hara [7]	17M	Sig	4	Early (M)	Not performed	Autopsy	Autopsy (microscopy on multiple sections)	Bilateral hilar lymphadenopathy	Details unknown
4	Yasui [8]	56M	Sig	15	Early (M)	Normal	Biopsy of cervical lymph node	Autopsy (microscopy on multiple sections)	Elevated tumor markers; cervical, mediastinal, hilar, and abdominal lymphadenopathy	CBDCA + PTX (death on the next day after CTx started)
5	Sato [6]	43M	Sig	ND	Early	ND	ND	ND	ND	AC
6	Sato [6]	68F	Sig	ND	Early	ND	ND	ND	ND	AC, etc. (details unknown)
7	Sato [6]	74M	Por + tub	ND	Early	ND	ND	ND	ND	CTx (details unknown)
8	Sato [6]	64F	Sig	ND	Early	ND	ND	ND	ND	Details unknown
9	Sato [6]	62F	Sig	ND	Early	ND	ND	ND	ND	Details unknown
10	Sato [6]	50M	Por + sig	ND	Early	ND	ND	ND	ND	CTx (details unknown)
11	Sato [6]	51F	Sig + tub + pap	ND	Early	ND	ND	ND	ND	Details unknown
12	Tateishi [9]	75M	Sig	1.8	Advanced	Normal	TBNA of mediastinal lymph node, TBLB	Autopsy (microscopy on multiple sections)	Mediastinal lymphadenopathy	CBDCA + PTX; AC (death on the 28th day after CTx started)
13	Present case	50M	Sig	15, 8	Early (M)	Normal	Needle biopsy of cervical lymph node	Autopsy (microscopy on multiple sections)	Elevated tumor markers; cervical, mediastinal, hilar, and upper abdominal lymphadenopathy	SOX; imatinib; AC; CS (death on the next day after SOX and imatinib started)

*PTTM* pulmonary tumor thrombotic microangiopathy, *SM* submucosa, *M* mucosa, *5-FU* 5-fluorouracil, *MMC* mitomycin C, *CDDP* cisplatin, *AC* anticoagulation, *ND* no data, *CTx* chemotherapy, *sig* signet ring cell carcinoma, *por* poorly differentiated adenocarcinoma, *tub* tubular adenocarcinoma, *pap* papillary adenocarcinoma, *TBNA* transbronchial needle aspiration, *TBLB* transbronchial lung biopsy, *CBDCA* carboplatin, *PTX* paclitaxel, *SOX* tegafur/gimeracil/oteracil plus oxaliplatin, *CS* corticosteroid

All reported cases of PTTM in early or micro-occult gastric cancer confirmed using autopsy had poorly cohesive histology, and almost all of them involved SRCC [3–9]. Poorly cohesive gastric carcinoma has a high tendency for lymphovascular invasion; hence, a large number of cancer cells flow into the pulmonary circulation via the thoracic duct. This is considered one of the main reasons that gastric cancer is the dominant cause of PTTM [6]. Similarly, early gastric cancer cases with Krukenberg tumor due to SRCC have been reported, and lymphatic metastasis is considered as the main pathway for the spread [10]. These reports suggest that despite the microscopic primary cancer lesion, as noted in Krukenberg tumor cases, gastric cancer with undifferentiated histology can progress to PTTM because of its tendency to include lymphovascular invasion. Recent studies have reported that early gastric SRCC, despite classification as undifferentiated histology, has a low risk of lymph node metastasis and a comparable prognosis with differentiated cancer [11, 12]. In addition, small size (less than 20 mm) and intramucosal lesions have a lower risk of lymph node metastasis [13]. Hence, micro-occult gastric SRCC presenting with PTTM, as listed in this case review, is considered rarer. However, lymphovascular invasion is also considered an independent risk factor of lymph node metastasis in early gastric SRCC, which indicates that micro-intramucosal SRCC can cause lymphatic invasion, although infrequently [13]. In contrast, definite risk factors of PTTM or Krukenberg tumor, which are considered a distinctive manifestation of lymphatic invasion in gastric cancer, are unknown. Future molecular and genetic research is, therefore, desirable in order to understand the pathogenesis of cases with early gastric cancer progressing to PTTM or Krukenberg tumor.

Of note, cases of PTTM due to SRCC in non-gastric cancer have occasionally been reported; conversely, no case of micro-occult cancer has been described in previous reports [14–17]. On the other hand, cases of PTTM due to SRCC of unknown primary origin should initially be suspected to be caused by gastric cancer. One autopsy case reported PTTM of SRCC probably due to gastric cancer without any mucosal lesions [18], and other reports described PTTM of SRCC of an unknown primary site with or without Krukenberg tumor [19–21]. However, in these cases, micro-occult gastric cancer may have been detected on autopsy if multiple serial sections of the whole stomach had been examined. Moreover, one PTTM case of SRCC of unknown primary origin was administered tegafur/gimeracil/oteracil plus cisplatin for presumptive gastric cancer despite normal endoscopic findings, similar to our case, and the patient survived for 15 months after diagnosis [22].

We made a pathological diagnosis of cancer using needle biopsy of the cervical lymph node. In one PTTM case of occult gastric cancer, which is not listed in Table 1 because of a lack of information about pathological invasion depth, multiple random endoscopic biopsies of the grossly normal gastric mucosa revealed SRCC [23]. A similar case has been reported for a Krukenberg tumor [24]. Thus, random gastric biopsy may be considered if PTTM of occult gastric cancer is suspected.

We initiated SOX therapy and imatinib on day 7; however, the patient died on day 8. The definitive treatment for PTTM is uncertain. Specific chemotherapy for a primary cancer is one of the treatment components; however, in many cases, it is not delivered because of the rapid clinical progression of the disease. Moreover, the efficacy of anticoagulation, vasodilators, or corticosteroids is unknown. Recent studies have reported the efficacy of imatinib, a platelet-derived growth factor (PDGF) receptor-tyrosine kinase inhibitor, with or without specific chemotherapy [25–31]. PDGF is one of the main humoral factors that stimulates fibrocellular intimal proliferation, leading to pulmonary hypertension in PTTM. Imatinib inhibits PDGF-receptor and PDGF release from cancer cells and plays a potential role in alleviating pulmonary hypertension in PTTM [1, 2]. Thus, although specific chemotherapy could not be delivered until the final pathological report, early administration of imatinib when PTTM is suspected (i.e., on day 1) might be an option. A prospective randomized study for optimal PTTM treatment is desired but practically difficult to carry out because of disease rarity, its rapidly progressive course, and ethical considerations.

In conclusion, physicians should suspect PTTM when encountering acute/subacute pulmonary hypertension without apparent etiology. Gastric cancer is the dominant primary origin; moreover, micro-occult gastric cancer can cause PTTM despite normal endoscopic findings. Taking prompt action is needed when PTTM is suspected, even if the patient appears stable.

## Supplementary Information

**Additional file 1.** **A** Fixed sample of the stomach shows no macroscopic abnormal mucosal lesions (oral side at the left, anal side at the right). **B** Red lines show the regions of the gastric cancer that were microscopically detected (oral side at the left, anal side at the right). **C** Red lines show the regions of the gastric cancer and green lines show lymphovascular invasion in multiple serial step sections of the whole stomach (oral side at the left, anal side at the right).

## Data Availability

Not applicable.

## Abbreviations

CDX-2: Caudal-type homeobox-2
CK: Cytokeratin
CT: Computed tomography
CTEPH: Chronic thromboembolic pulmonary hypertension
HNF-4α: Hepatocyte nuclear factor-4α
PTTM: Pulmonary tumor thrombotic microangiopathy
SOX: Tegafur/gimeracil/oteracil plus oxaliplatin
SRCC: Signet ring cell carcinoma
TTF-1: Thyroid transcription factor-1
