# Supplementary figures and images for: Pulmonary tumor thrombotic microangiopathy in occult early gastric cancer that was undetectable on upper endoscopy: a case report and review of similar cases
Source: BMC Gastroenterol. 2021 Nov 10;21:423. doi: 10.1186/s12876-021-02009-8 (PMC8579618; doi:10.1186/s12876-021-02009-8)

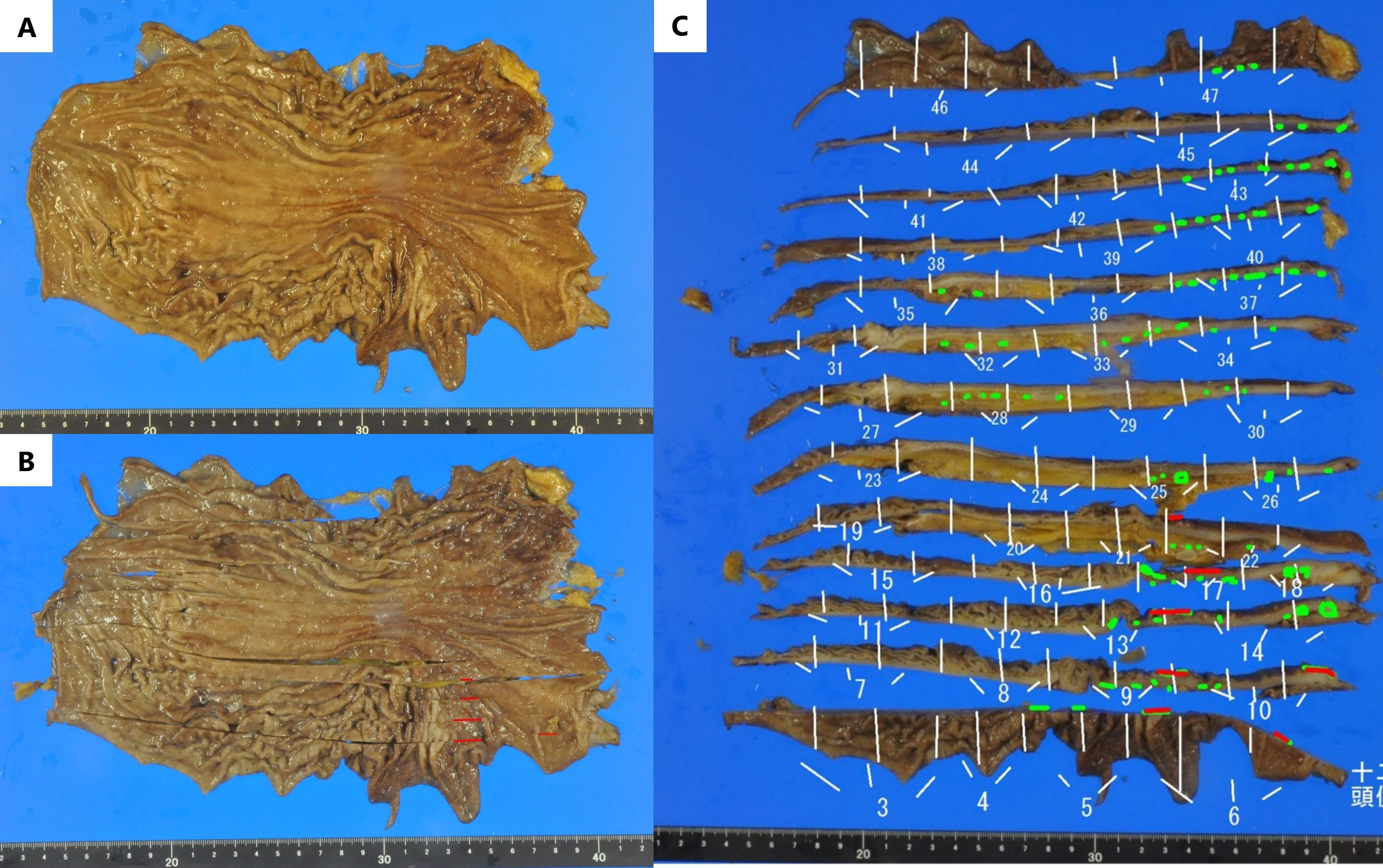

Supplement: Supplementary file 1 — Additional file 1. A Fixed sample of the stomach shows no macroscopic abnormal mucosal lesions (oral side at the left, anal side at the right). B Red lines show the regions of the gastric cancer that were microscopically detected (oral side at the left, anal side at the right). C Red lines show the regions of the gastric cancer and green lines show lymphovascular invasion in multiple serial step sections of the whole stomach (oral side at the left, anal side at the right). [file 12876_2021_2009_MOESM1_ESM.jpg]
